# Western Blotting and Immunoprecipitation of Native Human PIEZO1 Channels

**DOI:** 10.21769/BioProtoc.5385

**Published:** 2025-07-20

**Authors:** Jinyuan Vero Li, Zijing Zhou, Charles D. Cox

**Affiliations:** 1 Molecular Cardiology and Biophysics Division, Victor Chang Cardiac Research Institute, Sydney, New South Wales, Australia; 2 St Vincent's Clinical School, Faculty of Medicine, University of New South Wales, Sydney, New South Wales, Australia; 3 School of Biomedical Sciences, University of New South Wales, Sydney, New South Wales, Australia

**Keywords:** Mechanotransduction, Cross-linking prevention, Mechanosensitive channel, Auxiliary subunits, PIEZO1 Binding partners

## Abstract

PIEZO1 is a mechanically activated ion channel essential for mechanotransduction and downstream signaling in almost all organ systems. Western blotting is commonly used to study the expression, stability, and post-translational modifications of proteins. However, as a large transmembrane protein, PIEZO1 contains extensive hydrophobic regions and undergoes post-translational modifications that increase its propensity for nonspecific protein–protein interactions. As a result, conventional sample preparation methods seem unsuitable for PIEZO1. For example, heating and sonicating transmembrane proteins exposes hydrophobic regions, leading to aggregation, improper detergent interactions, and loss of solubility, ultimately compromising their detection in western blots. To address these challenges, we developed a western blot protocol optimized for human PIEZO1 by preparing lysates consistently at lower temperatures and incorporating strong reducing and alkylation reagents into the western blot lysis buffer to ensure proper protein solubilization and minimal cross-linking. Using the same antibody, we also developed an immunoprecipitation protocol with optimized detergents to maintain the solubilization of native human PIEZO1, enabling the discovery of a new family of auxiliary subunits.

Key features

• Simple modifications to the standard RIPA buffer prevent protein aggregates of large transmembrane proteins.

• Minimal protein degradation and cross-linking by modifying cell lysis conditions and protein extraction process.

• Clear separation of glycosylated and non-glycosylated PIEZO1 by SDS-PAGE.

## Background

PIEZO1 is a mechanically activated ion channel that responds to shear stress and membrane tension [1] to regulate downstream signaling pathways crucial in vascular development [2], cardiac remodeling [3], baroreception, and many other processes. Given its physiological importance, the ability to accurately detect and quantify PIEZO1 protein levels in cells and tissues is critical for studying its regulation and function. Western blotting is a widely used technique to examine protein expression, stability, and post-translational modifications (PTMs). For PIEZO1, western blotting is particularly useful for assessing PTMs such as ubiquitination or N-glycosylation [4], protein–protein interactions [5], and expression changes in response to cellular interventions. However, due to the unique structural properties of PIEZO1, traditional western blot methodologies seem inadequate for its detection.

To overcome these limitations, we have developed western blot and immunoprecipitation protocols specifically optimized for human PIEZO1. For western blot, we made key modifications that improve electrophoretic mobility and prevent cross-linking, degradation, and aggregation. We validated a monoclonal antibody from Novus Biologicals that gives robust human and pig PIEZO1 signals and recognizes both denatured and solubilized human PIEZO1. Importantly, using this protocol, this primary PIEZO1 antibody *does not* recognize mouse PIEZO1[4]. We make use of gradient polyacrylamide gels to enhance the separation of the large membrane proteins and enable effective transfer of human PIEZO1. This is coupled with optimized transfer conditions using extended time at a high stable current (140 min wet transfer at 300 mA). Furthermore, we used a strong reducing agent (TCEP) and an alkylation reagent (NEM) during cell lysis. Adding these two reagents allows PIEZO1 to yield a clear, robust band on western blot with repeated freeze-thaw cycles of the lysates. Instead of boiling at 95 °C, we prepare lysates continuously on ice before loading them onto the gel to prevent heat-induced protein aggregation. The only time we heat PIEZO1 samples is to remove immunoprecipitated human PIEZO1 from magnetic beads coated with the primary antibody. This immunoprecipitation protocol has ultimately allowed us to co-immunoprecipitate native human PIEZO1 along with associated proteins under near-native conditions and identify a new family of PIEZO channel auxiliary subunits.

## Materials and reagents

**Biological materials**

1. LNCaP clone FGC cell line [American Type Culture Collection (ATCC), catalog number: CRL-1740, derived from a metastatic prostate carcinoma]

2. HeLa cell line (ATCC, catalog number: CCL-2, derived from a cervical carcinoma)

3. MCF-7 cell line (ATCC, catalog number: HTB-22, from the pleural effusion of a 69-year-old Caucasian woman with metastatic breast adenocarcinoma)

4. HEK293T (ATCC, catalog number: CRL-3216, a derivative of the original HEK293 cells stably expressing SV40 large T antigen)

5. HEK293T-*Piezo1-/-* (gift from Ardem Patapoutian)

6. Human dermal fibroblasts BJ-5ta cell line (ATCC, catalog number: CRL-4001, hTERT-immortalized human foreskin fibroblast line, derived from a neonatal male)

7. Human cardiac fibroblasts (HCF) (PromoCell GmbH, catalog number: C-12375, isolated from the ventricles of adult human hearts)

8. Porcine aortic endothelial cells (PAOEC) (Cell Applications, Inc., catalog number: P304-05, isolated from normal healthy porcine aorta)

9. Human umbilical vein endothelial cells (HUVECs) (ATCC, catalog number: PCS-100-010, isolated from the umbilical vein of human umbilical cords)

**Reagents**

1. Dulbecco's modified Eagle medium (DMEM) (Sigma-Aldrich, catalog number: D6429)

2. RPMI 1640 medium with L-Glutamine (Gibco, catalog number: 23400062)

3. EGM^TM^-2 Endothelial Cell Growth Medium-2 BulletKit^TM^ (Lonza Bioscience, catalog number: CC-3162)

4. FGM^TM^-2 Fibroblast Growth Medium-2 BulletKit^TM^ (Lonza Bioscience, catalog number: CC-3132)

5. HyClone^TM^ characterized fetal bovine serum (FBS), Australian origin (Cytiva, catalog number: DE29445779)

6. TrypLE^TM^ Express trypsin (Gibco, catalog number: 12604021)

7. Dulbecco’s phosphate-buffered saline (DPBS) (Gibco, catalog number: 14190144)

8. Sodium hydroxide (NaOH) (Sigma-Aldrich, CAS number: 1310-73-2)

9. Ethylenediaminetetraacetic acid (EDTA) (Sigma-Aldrich, CAS number: 60-00-4)

10. Sodium deoxycholate (Sigma-Aldrich, CAS number: 302-95-4)

11. Sodium dodecyl sulfate (SDS) (Sigma-Aldrich, CAS number: 151-21-3)

12. Triton X-100 (Thermo Scientific^TM^, catalog number: 85112)

13. Tris (2-carboxyethyl) phosphine (TCEP) hydrochloride (Sigma-Aldrich, CAS number: 51805-45-9)

14. N-ethylmaleimide (NEM) (Thermo Scientific, catalog number: 23030)

15. Phenylmethylsulfonyl fluoride (PMSF) (Roche, CAS number: 329-98-6)

16. cOmplete^TM^ EDTA-free mini protease inhibitor cocktail tablets (Roche, catalog number: 11836170001)

17. Polyethylene glycol sorbitan monolaurate (TWEEN^®^ 20) (Sigma-Aldrich, CAS number: 9005-64-5)

18. TRIS-buffered saline (TBS, 10×) pH 7.4 (Thermo Fisher Scientific, catalog number: J60764.K7)

19. Sodium azide (NaN_3_) (Sigma-Aldrich, catalog number: S2002)

20. Pierce^TM^ BCA Protein Assay kit, reducing agent compatible (Thermo Fisher Scientific, catalog number: 23250)

21. PIEZO1 antibody (Clone 2-10) (Novus Biologicals, catalog number: NBP2-75617)

22. Goat anti-mouse IgG secondary antibody, IRDye 800CW (LI-COR Biosciences, catalog number: 926-32210)

23. α-ACTININ (H-2) antibody (Santa Cruz Biotechnology, catalog number: sc-17829)

24. Dynabeads^TM^ protein G for immunoprecipitation (ThermoFisher, catalog number: 10003D)

25. Soy PC (95%) (Avanti, catalog number: 441601G)

26. CHAPS hydrate (Sigma-Aldrich, CAS number: 331717-45-4)

27. PIPES (Sigma-Aldrich, CAS number: 5625-37-6)

28. Urea (QIAGEN, catalog number: 11557305)

29. Millex^TM^ MCE syringe filter (Millipore, catalog number: SLGSR33SS)

30. NuPAGE^TM^ tris-acetate mini protein gels, 3%–8%, 1.0 mm, 10 wells, 10 gels/box (Invitrogen, catalog number: EA0375BOX)

31. UltraPure™ glycerol (ThermoFisher, catalog number: 15514011)

32. Bromophenol blue powder (Bio-Rad, catalog number: 1610404)

33. Tris(hydroxymethyl)aminomethane (Sigma-Aldrich, CAS number: 252859)

34. Ponceau S (Sigma-Aldrich, CAS number: 141194)

35. Glacial acetic acid (Sigma-Aldrich, CAS number: A6283)

36. Sodium piperazine-N, N′-bis (2-ethanesulfonic acid) (NaPIPES) (Sigma-Aldrich, CAS number: P2949)

37. Soy phosphatidylcholine (Sigma-Aldrich, CAS number: P7443)

38. DL-Dithiothreitol (ThermoFisher, catalog number: R0861)

**Solutions**

1. 200 mM PMSF in ethyl alcohol (see Recipes)

2. 2 M NEM in ethyl alcohol (see Recipes)

3. 1 M TCEP in water (see Recipes)

4. 1× Modified radio-immunoprecipitation assay (RIPA) buffer (see Recipes)

5. 5× Sample loading buffer (see Recipes)

6. Running buffer (see Recipes)

7. Transfer buffer (see Recipes)

8. Ponceau S Solution (see Recipes)

9. Immunoprecipitation lysis buffer (see Recipes)

10. Immunoprecipitation wash buffer (see Recipes)

11. Immunoprecipitation elution buffer (see Recipes)

**Recipes**

**1. 200 mM PMSF in ethyl alcohol (store at -20 °C)**

**Table:** 

Reagent	Final concentration	Quantity
PMSF	200 mM	35 mg
Pure ethanol	n/a	1 mL
Total	n/a	1 mL

**2. 2 M NEM in ethyl alcohol (store at -20 °C)**

**Table:** 

Reagent	Final concentration	Quantity
NEM	2 M	222.28 mg
Pure ethanol	n/a	1 mL
Total	n/a	1 mL

**3. 1 M TCEP in water (store at -20 °C)**

**Table:** 

Reagent	Final concentration	Quantity
TCEP hydrochloride	1 M	286.65 mg
Distilled H_2_O	n/a	684 μL
10 M NaOH solution	Adjust pH to 7	316 μL
Total	n/a	1 mL

**4. 1× Modified radio-immunoprecipitation assay (RIPA) buffer (store at -20 °C)**

**Table:** 

Reagent	Final concentration	Quantity
Distilled water		9550 μL
1 M Tris buffer (pH 7.5)	10 mM	100 μL
EDTA	1 mM	3.7224 mg
NaCl	140 mM	81.82 mg
Sodium deoxycholate	0.1% w/v	10 mg
SDS	0.1% w/v	10 mg
Triton X-100	1% v/v	100 μL
200 mM PMSF	1 mM	50 μL
1 M TCEP (pH 7)	10 mM	100 μL
2 M NEM	20 mM	100 μL
EDTA-free mini protease inhibitor cocktail tablets	1×	1 tablet
Total	n/a	10 mL

TCEP and NEM can react [6], which may impair the reducing and alkylating efficiency. One alternative option to circumvent this is to first lyse cells using modified RIPA buffer without NEM, then add 20 mM NEM to prevent the potential TCEP-NEM reaction.

Additionally, 25 mM sodium fluoride (NaF) and 1 mM sodium orthovanadate (NaVO_3_) can be added to the modified RIPA if there is a necessity to investigate phosphorylated proteins.

**5. 5× sample loading buffer (store at room temperature)**

**Table:** 

Reagent	Final concentration	Quantity
SDS	10% w/v	10 g
Glycerol	50% v/v	50 mL
1 M Tris-HCl (pH 6.8)	250 mM	25 mL
Bromophenol blue	500 μM	33.5 mg
Distilled H_2_O	n/a	25 mL
Total	n/a	100 mL

**6. Running buffer (store at room temperature)**

**Table:** 

Reagent	Final concentration	Quantity
Glycine	1.5% w/v	15 g
Tris(hydroxymethyl)aminomethane	0.5% w/v	5 g
SDS	0.1% w/v	1 g
NaOH	0.3% w/v	3 g
Distilled H_2_O	n/a	1 L
Total	n/a	1 L

**7. Transfer buffer (store at room temperature)**

**Table:** 

Reagent	Final concentration	Quantity
Glycine	1% w/v	10 g
Tris(hydroxymethyl)aminomethane	0.25% w/v	2.5 g
Absolute ethanol	20% v/v	200 mL
Distilled H_2_O	n/a	800 mL
Total	n/a	1 L

**8. Ponceau S Solution (store at room temperature)**

**Table:** 

Reagent	Final concentration	Quantity
Ponceau S Red	0.1% w/v	100 mg
Glacial acetic acid	5% v/v	5 mL
Distilled H_2_O	n/a	95 mL
Total	n/a	100 mL

**9. Immunoprecipitation lysis buffer (store at -20 °C)**

**Table:** 

Reagent	Final concentration	Quantity
Distilled water		10 mL
Sodium piperazine-N,N′-bis(2-ethanesulfonic acid) (NaPIPES)	25 mM, pH 7.2	75.6 mg
EDTA	1 mM	3.71 mg
CHAPS	1% w/v	100 mg
NaCl	140 mM	81.82 mg
Soy phosphatidylcholine	0.6% w/v	60 mg
DTT	2 mM	3.09 mg
EDTA-free mini protease inhibitor cocktail tablets	1×	1 tablet
Total	n/a	10 mL

**10. Immunoprecipitation wash buffer (store at -20 °C)**

**Table:** 

Reagent	Final concentration	Quantity
Distilled water		10 mL
NaPIPES	25 mM, pH 7.2	75.6 mg
EDTA	1 mM	3.71 mg
CHAPS	0.5% w/v	50 mg
NaCl	140 mM	81.82 mg
Soy phosphatidylcholine	0.14% w/v	28 mg
DTT	2 mM	3.09 mg
EDTA-free mini protease inhibitor cocktail tablets	1×	1 tablet
Total	n/a	10 mL

**11. Immunoprecipitation elution buffer (store at -20 °C)**

**Table:** 

Reagent	Final concentration	Quantity
5× sample loading buffer	1×	200 μL
8 M urea	1 M	125 μL
1 M TCEP	10 mM	10 μL
Distilled H_2_O	n/a	665 μL
Total	n/a	1 mL

**Laboratory supplies**

1. Corning^®^ 25cm^2^ rectangular canted neck cell culture flask with vented cap (Corning Incorporated, catalog number: 431463)

2. Costar^®^ 24-well clear TC-treated 24-well plates (Corning Incorporated, catalog number: 3524)

3. QSP snap cap microcentrifuge tubes (Thermo Scientific, catalog number: 509-GRD-Q)

4. Corning^®^ Costar^®^ Stripette^®^ serological pipette (Corning Incorporated, catalog number: CLS4488)

5. Novex^TM^ sharp pre-stained protein standard (Invitrogen, catalog number: LC5800)

## Equipment

1. LI-COR Odyssey 9120 Infrared Imaging System (LI-COR Biosciences, model: 9120)

2. Heracell 150 CO_2_ Incubator (Thermo Fisher Scientific, model: 51026283)

3. Eppendorf Centrifuge 5425 R (Eppendorf AG, model: EP5406000569)

4. TE 22 Mini Tank Transfer Unit (Cytiva, model: 80620426)

5. Cassette with sponges TE22 Mini Tank Transfer Unit (Hoefer, Inc. catalog number: TE24)

6. Whatman^TM^ Grade 3MM Chr chromatography paper (Cytiva, catalog number: 3030-917)

7. Nitrocellulose membrane, 0.2 μm (Bio-Rad Laboratories, catalog number: 1620112)

8. Refrigerated water bath with pump out facility (10 Litre) (Ratek Instruments Pty Ltd. model: BL-30)

9. PowerPac^TM^ basic power supply (Bio-Rad Laboratories, catalog number: 164-5050)

10. XCell SureLock^TM^ Mini-Cell gel electrophoresis system (Invitrogen^TM^, catalog number: EI0001)

11. PHERAstarFS microplate reader (BMG LABTECH, model: FSX)

12. Bright-Line^TM^ hemacytometer (Hausser Scientific, model: Z359629)

13. DynaMag^TM^-2 magnet (ThermoFisher, catalog number: 12321D)

## Software and datasets

1. Image Studio^TM^ Software (LI-COR Biosciences, versions 4.x)

2. Microsoft Excel (Microsoft, Microsoft Office 2020)

## Procedure

**A. Prepare cells for protein extraction**

1. Culture HEK293T, MCF-7, human dermal fibroblasts (BJ-5ta), and HeLa cells in DMEM supplemented with 10% v/v FBS. Culture LNCaP in RPMI-1640 supplemented with 10% v/v FBS. Culture HUVEC and PAOEC in EGM^TM^-2, and HCF in FGM^TM^-2. Incubate all cell lines at 37 °C with 5% CO_2_ in a humidified incubator in T25 flasks. Cells display differential PIEZO1 N-glycosylation, which can be investigated in the western blots generated using this protocol.

2. Gently wash the cells with 5 mL of 25 °C DPBS, then discard the DPBS completely before adding 500 μL of trypsin (TrypLE^TM^ Express) to the cells.

3. Gently agitate culture flasks to ensure trypsin is evenly spread on the surface before incubating at 37 °C for 3–5 min.

4. Add 2 mL of culture medium to the flasks to suppress trypsin digestion and do 3–5 rounds of rigorous pipetting to separate all cell clusters.

5. Inject 10 μL of suspension into a Bright-Line^TM^ hemacytometer to calculate the cell density.

6. After calculating cell density, pipette cell suspension rigorously twice, then add a volume containing ~25 K cells into one well of a 24-well plate. Supplement the culture medium for the relevant cell lines into the 24-well plate to make up a final volume of 1 mL before putting the 24-well plate back into the incubator overnight.

7. For immunoprecipitation, the number of cells required depends on the native expression level of PIEZO1. In our experience with human dermal fibroblasts, a 60 or 100 mm dish of cells at a confluence of ~80% will give a measurable PIEZO1 signal. If mass spectrometry profiling is planned, prepare at least 2 × 150 mm dishes for each condition.

**B. Extraction of proteins from cells for western blotting**

1. Prepare modified 1× RIPA buffer on ice or thaw frozen buffer prepared previously, pre-cool a 5425 R centrifuge to 4 °C, and pre-cool tubes on ice.

2. Place the 24-well plate containing cells on ice. Discard all culture media and then add 100 μL of modified 1× RIPA buffer into each well. If necessary, scrape cells using the back of a 10 μL pipette tip to release all cells into the RIPA buffer.

3. Put the 100 μL modified 1× RIPA into a 1.5 mL microcentrifuge tube pre-cooled on ice.

4. Spin the lysate at 13,000× *g* for 5 min at 4 °C. Collect the supernatant and place it into another pre-cooled 1.5 mL microcentrifuge tube on ice for 30 min.

**C. Extraction of proteins from cells for immunoprecipitation**

1. Prepare fresh 1× immunoprecipitation lysis buffer and pre-cool a 5425 R centrifuge to 4 °C.

2. Place the 60 mm dish containing cells on ice. Discard all culture media, wash with cold PBS once, and then add 500 μL of ice-cold immunoprecipitation lysis buffer into the 60 mm dish. For immunoprecipitation coupled with downstream mass spectrometry (IP-MS), use 2 mL of modified immunoprecipitation lysis buffer to lyse the cells from both 150 mm dishes after washing with PBS.

3. Lyse the cells on ice for at least 30 min before centrifugation.

4. Spin the lysate at 13,000× *g* for 10 min at 4 °C. Collect 450 μL of supernatant and place it into another pre-cooled 1.5 mL QSP tube. For IP-MS, collect 1.9 mL of supernatant and add to a 15 mL Falcon tube.

5. From the supernatant, freeze 20 μL of lysate at -80 °C to use as the *input* fraction. This step can be skipped for IP-MS.

6. Keep the remaining lysate on ice. Mix 10 μL of protein G Dynabeads with 1 µg of PIEZO1 antibody and 430 μL of the cell lysate from step C4 and then incubate on a rotator at 4 °C overnight. For the larger-scale immunoprecipitation for IP-MS, add 30 μL of protein G Dynabeads with 6 μg of PIEZO1 antibody to the 1.9 mL of lysate.

7. Prepare fresh 1× immunoprecipitation wash buffer on ice.

8. Place the tubes with the lysate–Dynabeads mixture onto a DynaMag^TM^-2 magnet and let the magnetic beads attach for 30 s.

9. Remove the supernatant and wash the beads by adding 500 μL (or 1 mL, for IP-MS) of immunoprecipitation wash buffer per tube. Place the tubes back on the rotator at 4 °C to wash for 10 min. Repeat this step three times.

10. Finally, remove the wash buffer and add 20 μL of ice-cold 1× immunoprecipitation elution buffer to the beads. Heat the beads at 95 °C for 5 min (for immunoprecipitation samples that will undergo mass spectrometry) or 60 °C for 5 min (for western blot pipelines). This eluted fraction becomes the *IP* fraction.

11. The input and IP fractions are loaded at equal volumes for the western blot analysis.

*Note: For immunoprecipitation coupled with mass spectrometry, two extra steps are necessary for high-quality data. First, a pre-clearing step is performed where 30 μL of protein G Dynabeads is mixed with the supernatant at 4 °C overnight. The mixture is centrifuged at 13,000× g for 10 min at 4 °C, and the pellet is discarded. Second, the supernatant collected from the first step is filtered with a Millex^TM^ MCE syringe filter (0.22 μm, diam. 33 mm, sterile, hydrophilic). The filtered supernatant is then subjected to step C6 onward.*

**D. Total protein quantification**

1. From the supernatant, 2 μL of lysate can be taken for total protein calculation. For IP experiments, this step can be skipped.

2. Note that because of the modified RIPA, this requires a reducing reagent–compatible kit such as the Pierce^TM^ BCA Protein Assay kit. Follow the manufacturer’s instructions and finally measure absorbance at 562 nm using a PHERAstarFS microplate reader.

3. Afterward, mix 10–30 μg of total protein from each sample with 20% volume of 5× sample loading buffer on ice before loading onto a gel.

4. Note that for a 1–1.5 mm 10-well gel, the final sample volume cannot exceed 40 μL, and for a 15-well gel, the final volume cannot exceed 15 μL.

**E. Electrophoresis**

1. Load a 15-well 3%–8% tris-acetate gel into a mini-cell gel tank.

2. Pour running buffer into the tank to cover the gel wells at the top and the opening portion at the bottom. Note that other gradient gels, such as NuPAGE^TM^ bis-tris mini protein gels, 4%–12%, 1.0–1.5 mm (Invitrogen), can also be used.

3. Load samples and 5 μL of sharp pre-stained protein standard ladder into each well of the gel slowly. (Make sure your pipette tip reaches just above the bottom of the well before releasing.)

4. After sample loading, connect the tank to a PowerPac and set it to a constant voltage for electrophoresis. Run at 80 V for 30 min, then 110 V for 90 min until the dye front reaches the end of the gel.

**F. Transferring**

1. For transferring, we found wet transfer was the most efficient for human PIEZO1 protein. For this, we use a TE22 mini tank connected to a refrigerated water bath with a pump, where the tank is placed on a magnetic stirrer.

2. Pour the transfer buffer into the TE22 mini tank until the liquid surface reaches 1 cm below the max line.

3. Switch on the refrigerated pump and magnetic stirrer. These preparation steps aim at pre-cooling the transfer buffer, which we find is essential for efficient transfer.

4. Cut nitrocellulose membrane and chromatography paper into necessary sizes (we cut the membrane into 6 × 7.5 cm and filter paper into 9 × 9 cm) and put them together with the cassette and sponges of the TE22 mini tank transfer unit. Then, immerse all items in the transfer buffer at room temperature. This is to avoid gas bubbles forming between the gel and the membrane in the following transferring process.

5. When electrophoresis is finished, pry open the gel cage and take out the gel with the 2 mm bottom part trimmed.

6. Make a sandwich structure in the transfer buffer in a sponge-paper-gel-membrane-paper-sponge order.

7. Carefully avoid gas bubbles stuck between the gel and the membrane.

8. Stabilize the sandwich using the cassette, then insert the sandwich into the TE22 transfer tank and immerse it into the pre-cooled transfer buffer.

9. Connect the TE22 to a PowerPac, which is set to a constant current mode, running at 300 mA for 140 min, with the cooling system continuously on.

**G. Transfer quality control and membrane blocking**

1. After 140 min, when the transfer is finished, the removed membrane can be stained with Ponceau S solution to check the transfer efficiency and quality.

2. For Ponceau S staining, cover the protein side of the membrane with 2 mL of Ponceau S solution for 1 min; then, wash away the excess Ponceau S solution using tap water. Protein bands are then stained red and can also be used as total protein normalization. Patchy stains or invisible protein bands are indicative of a failed transfer. Ponceau S-stained protein bands also facilitate transverse membrane segmentation for probing different regions of the membrane using different antibodies.

3. For membrane blocking, use 300 mg of skim milk powder dissolved in 10 mL of TBS buffer. Then, pour the resulting blocking buffer onto the membrane and incubate on an orbital shaker at 60–80 rpm at 4 °C overnight or at room temperature for 1 h.

**H. Primary antibody incubation**

1. Discard the blocking buffer and wash the membrane using tap water.

2. Apply the Novus PIEZO1 antibody and Santa Cruz α-ACTININ primary antibody (or other relevant loading control for the cell of choice) diluted 1:1,000 in 5 mL of TBS buffer.

3. Incubate the membrane in the diluted antibodies on a rocker for 1 h at room temperature or overnight at 4 °C (especially in cells with low PIEZO1 expression).

**I. First membrane washing step**

1. Wash the membrane in 10 mL of TBS supplemented with 0.1% v/v TWEEN^®^ 20 on an orbital shaker rotating at 80 rpm at room temperature twice, each time for 10 min.

2. Note that the used diluted primary antibody (Novus PIEZO1 antibody) can be stored at -20 °C and reused for two further rounds of western blotting, if desired.

**J. Secondary antibody incubation**

1. We prefer to utilize fluorophore-conjugated secondary antibodies, but the protocol will work with horseradish peroxidase-conjugated secondary antibodies for classical western blot pipelines. Our preference is for a goat anti-mouse secondary antibody diluted 1:10,000 in 10 mL of TBS supplemented with 0.02% w/v sodium azide.

2. Apply the secondary antibody dilution to the membrane, which is then incubated on an orbital shaker at 60 rpm at room temperature for 40 min.

**K. Second membrane washing step**

1. After secondary antibody incubation, wash the membrane using 10 mL of TBS supplemented with 0.1% v/v TWEEN^®^ 20 on an orbital shaker rotating at 80 rpm at room temperature twice, each time for 10 min.

2. Again, the used diluted secondary antibody can be stored at 4 °C and reused multiple times within a month.

3. It is recommended to immediately scan the membrane. If not, store the membrane in 1× TBS buffer at 4 °C for no more than one week.

**L. Signal detection using LI-COR Odyssey**

1. Ensure the scanning surface of the LI-COR Odyssey scanner is clean using distilled water. The membrane should remain moist in distilled water, and the protein side should be laid down on the scanning surface. Bubbles between the scanning surface and the membrane should be avoided.

2. Depending on the secondary antibodies used, scan the membrane with either 700 nm (red, for IRDye 680) or 800 nm (green, for IRDye 800). Scan parameters can then be selected that enable visualization of not only the secondary antibody signal but also the bands from the pre-stained protein ladder for equivalent size determination.

3. After the scan, the scan surface should be cleaned using 70% ethanol and then distilled water. No liquid should be left behind to avoid damage.

**M. Signal quantification**

**Figure 1.**
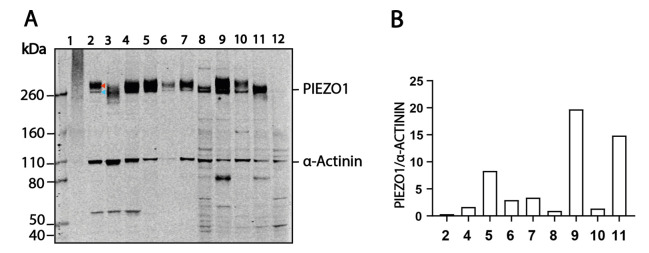
Representative western blot and expression level quantification between human and pig cell lines. (A) Representative western blot probing PIEZO1 and α-ACTININ from various cell lines. For protein lanes: 1, sonicated HCF; 2, HCF; 3, 95 °C boiled HCF; 4, HUVEC; 5, PAOEC; 6, HCF (high passage number); 7, BJ-5ta; 8, HEK293T; 9, MCF-7; 10, HeLa; 11, LnCaP; 12, HEK293T-*Piezo1-/-*. (B) Example of the quantified intensity level of PIEZO1 signals and α-ACTININ signals from multiple cell lines.

1. Import the scanned file into the Image Studio^TM^ Software. Adjust the angle and contrast using the *free rotate* tool and the *display* panel.

2. Select regions of interest (ROIs) by drawing rectangles in the *shape* tab.

3. Simple background subtraction can be done by clicking *Analysis, Background*, and then *Median* to subtract the median intensity of the surrounding area.

4. An identical ROI size should be used to measure signal intensity for the same protein in different lanes. The intensity values of ROIs are then displayed in the software beside the rectangle.

5. As an example, we show the intensity values of PIEZO1 and housekeeping gene α-ACTININ from each lane of a blot to normalize the expression level and account for variable loading in a variety of human cell lines (Figure 1).

In Figure 1, lanes 2 and 4–11 present the correct PIEZO1 size. The blots show a similar core-N-linked glycosylated PIEZO1 band (blue arrow, lane 2) at a lower molecular weight and a fully-N-linked glycosylated band (red arrow, lane 2) at a higher molecular weight. Different cell lines construct glycan chains of different sizes for the fully-N-linked glycosylation. As a result, the higher PIEZO1 band from various cell lines is of different sizes [4].

In comparison, lane 1 shows that sonication totally degraded PIEZO1 protein, and lane 3 clearly shows the high-temperature-induced protein degradation. Note that we tried heating PIEZO1 at multiple temperatures, including 60 °C, for different durations in our modified RIPA buffer. None of the heating conditions enabled PIEZO1 to show bands at the correct size in the western blot.

## Validation of protocol

This protocol (or parts of it) has been used and validated in the following research articles:

- Vero et al. [7]. The anchor domain is critical for Piezo1 channel mechanosensitivity. *Channels* (Figure 2b and Figure 3d)
- Vero et al. [4]. Modified N-linked glycosylation status predicts trafficking defective human Piezo1 channel mutations. *Communications Biology* (Figure 1a–b, d–h, j, Figure 2a, e–f, Figure 3c, Figure 5b, f, Figure 6c, g–i, Figure 7a, and Figure 8a–b).
- Zhou et al. [8]. Piezo1 Mutations Display Altered Stability Driven by Ubiquitination and Proteasomal Degradation. *Frontiers in Pharmacology* (Figure 1a, Figure 2a–c, and Figure 3a).
- Zhou et al. [5]. MyoD-family inhibitor proteins act as auxiliary subunits of Piezo channels. *Science* (Figure 1d and Supplementary Figure 1).

## General notes and troubleshooting

**General notes**

1. The nitrocellulose membrane is not supposed to be dry from the moment that it is moist to the end of signal scanning.

2. PIEZO1 may show two bands from certain cell lines. This is because PIEZO1 undergoes heavy N-linked glycosylation; so, in the conditions documented in this protocol, you can detect two distinct bands, especially in primary cells.

3. Different batches of lysate from the same line may show different normalized protein levels of PIEZO1. This is because the expression of PIEZO1 can be regulated by cell status (e.g., fibroblast activation level) and culture conditions (e.g., substrate stiffness).

**Troubleshooting**

Problem 1: “Gooey” lysate.

Possible cause: Release of genomic DNA in the lysate.

Solution: Keep the SDS concentration lower than 0.1% in the RIPA buffer to prevent damage to the nuclear envelope. To solve this problem, 2 µg/mL of DNAse I and 2.5 mM MgCl can be mixed into the gooey lysate and then incubated at 4 °C for 2 h before loading, instead of sonicating the lysate.

Problem 2: Patchy/uneven/weak Ponceau S stain after transfer.

Possible cause: Gas bubbles between the membrane and the gel, the sandwich was loose, or the transfer buffer was overheated or old.

Solution: Carefully avoid bubbles when putting the gel membrane together. Make sure the sandwich is tight, the tubing of the cooling system is not narrow, and the magnetic stirrer is spinning. Use new transfer buffer.

## References

[1] Cox C. D., Bae C., Ziegler L., Hartley S., Nikolova-Krstevski V., Rohde P. R., Ng C. A., Sachs F., Gottlieb P. A., Martinac B., .(2016). Removal of the mechanoprotective influence of the cytoskeleton reveals PIEZO1 is gated by bilayer tension. Nat Commun. 7(1): e1038/ncomms10366. 10.1038/ncomms10366 PMC4735864 26785635

[2] Li J., Hou B., Muraki K., Ainscough J. and Beech D. (2015). Piezo1 Integration of Vascular Architecture with Physiological Force. The FASEB Journal 29: e2. 10.1096/fasebj.29 .1_supplement.639.2 PMC4230887 25119035

[3] Yu Z. Y., Gong H., Kesteven S., Guo Y., Wu J., Li J. V., Cheng D., Zhou Z., Iismaa S. E., Kaidonis X., .(2022). Piezo1 is the cardiac mechanosensor that initiates the cardiomyocyte hypertrophic response to pressure overload in adult mice. Nat Cardiovasc Res. 1(6): 577 591 591. 10.1038/s44161-022-00082-0 39195867 PMC11358016

[4] Li J. V., Ng C. A., Cheng D., Zhou Z., Yao M., Guo Y., Yu Z. Y., Ramaswamy Y., Ju L. A., Kuchel P. W., .(2021). Modified N-linked glycosylation status predicts trafficking defective human Piezo1 channel mutations. Commun Biol. 4(1): e1038/s42003–021–02528–w. 10.1038/s42003-021-02528-w PMC8421374 34489534

[5] Zhou Z., Ma X., Lin Y., Cheng D., Bavi N., Secker G. A., Li J. V., Janbandhu V., Sutton D. L., Scott H. S., .(2023). MyoD-family inhibitor proteins act as auxiliary subunits of Piezo channels. Science. 381(6659): 799 804 804. 10.1126/science.adh8190 37590348

[6] Shafer D. E., Inman J. K. and Lees A. (2000). Reaction of Tris(2-carboxyethyl)phosphine(TCEP) with Maleimide and α-Haloacyl Groups: Anomalous Elution of TCEP by Gel Filtration. Anal Biochem. 282(1): 161 164 164. 10.1006/abio.2000 .4609 10860517

[7] Vero Li J., C. D Cox and Martinac B. (2021). The anchor domain is critical for Piezo1 channel mechanosensitivity. Channels. 15(1): 438 446 446. 10.1080/19336950.2021 .1923199 33975519 PMC8118467

[8] Zhou Z., Li J. V., Martinac B. and Cox C. D. (2021). Loss-of-Function Piezo1 Mutations Display Altered Stability Driven by Ubiquitination and Proteasomal Degradation. Front Pharmacol. 12: e766416. 10.3389/fphar.2021 .766416 PMC8640252 34867393

